# Sleep and Learning: A Systematic Review

**DOI:** 10.1055/s-0043-1777294

**Published:** 2024-02-05

**Authors:** Martha Lucía Gutiérrez Pérez, Juan Antonio Lugo Machado, Valeria Lozano Lavado, Diana Camila Navarro Pimiento

**Affiliations:** 1 Otorhinolaryngology Interest Group UEB (ORLIG-UEB), Colombian School of Medicine, Universidad El Bosque, Bogotá, Colombia; 2 Department of Head and Neck Surgery, Hospital de Especialidades núm. 2, Centro Médico Nacional del Noroeste, Instituto Mexicano del Seguro Social, Ciudad Obregón, Sonora, Mexico; 3 Hospital General de Obregón, Ciudad Obregón, Sonora, Mexico

**Keywords:** sleep, learning, internship and residency, systematic review

## Abstract

**Introduction** Sleep deprivation has a great impact on the learning process in physicians in training. Therefore, inquiring on this phenomenon in the most recent investigations will facilitate the provision of evidence on the influence regarding the absence of sleep on the learning process in health personnel.

**Objectives** The aim of this systematic review is to review, analyze and discuss the current literature that shows the impact of sleep on the learning process on doctors in training.

**Data Synthesis** A systematic review was performed according to the Preferred Reporting Items for Systematic Reviews and Meta-Analysis (PRISMA) guidelines. A search of the existing literature between the years of 2000 and 2022 was performed in the PubMed and Elsevier databases, taking into account the inclusion criteria of articles in English or Spanish and the established timeframe. As a result, 128 articles distributed in the databases were obtained and 23 articles that met the inclusion criteria were selected.

**Conclusion** Sleep is a fundamental factor for the consolidation, processing and functioning of memory and learning. Health professionals are a population at risk of sleep deprivation, thus it is important to take into account the effects it has on patients and health personnel.

## Introduction

Research by Fotuhi et al. and Carskadon et al. has revealed that adequate sleep is vital for optimal cognitive function throughout life. 1 2 Although the association between sleep and cognitive function is likely bidirectional, it has been suggested that alterations in sleep duration may occur before the onset of cognitive symptoms in the Alzheimer disease. 3

The function of sleep remains unknown despite our increasing understanding of the processes that generate and maintain it. Several hypotheses about nonmutually exclusive functions have been proposed: for example, energy conservation, 4 brain thermoregulation, 5 brain detoxification, 6 and tissue “restoration”. 7 Another hypothesis proposes that sleep periods are favorable for brain plasticity and, in adults, for learning and memory. 8

Some theories defend that sleep is mainly involved in the processing of memory fragments, which can be operationally described in three main steps: exposure to the new stimulus, memory processing, and performance on a new test. 8 The more conventional view is that sleep processes participate in the consolidation of memory traces. 8 Consolidation refers to the processing of memory traces, during which they “can be reactivated, analyzed, and gradually incorporated into long-term memory”. 9

Those brain activities during sleep that are dependent on the preceding waking period have been interpreted in two different ways: experience-dependent or use-dependent processes. 8 The latter reflects the restoration of optimal (essentially synaptic) neuronal function after sustained waking neuronal activity. 8 It does not assume any exposure to a new environment (stimulus, task), nor the expansion of the behavioral repertoire. 8 In humans, slow-wave activity during sleep has been shown to increase in the central area of the brain, contralateral to prolonged vibration hand stimulation experienced in the earlier wake phase. 10

### Common Mechanisms in the Formation of Memory and Sleep

The memory formation process is composed of two main phases: encoding and consolidation. 11 The encoding phase is associated with hippocampal long-term potentiation plasticity, involving the formation of new memory traces that are initially fragile and vulnerable to external influences. 11 Then, in the consolidation phase, a fragile memory trace is transferred to more permanent long-term storage in the neocortex for later retrieval. 12 Therefore, this last phase is also associated with systemic reorganization. 12

The importance of the consolidation phase has been explored through pharmacological and electrophysiological interventions administered in different time windows after learning. 13 Perception and processing of information during encoding and retrieval require an active and alert brain. 14 In contrast, skill consolidation occurs in the absence of attention and during sleep. 14

It is assumed that there is less interference from other stimuli during sleep, which protects the stabilization of a newly created memory fragment. 13 In addition to this passive role of sleep, a reactivation of memory representations in hippocampal and nonhippocampal areas through mechanisms of synaptic plasticity has been demonstrated in animal models and in human studies during the different phases of sleep, but with a predominance in slow sleep waves. 15 16

Two theoretical models have been proposed for these interactions between memory formation and sleep: the active system consolidation hypothesis and the synaptic homeostasis hypothesis. 17 The first model refers to the dialogue between the hippocampus and the neocortex, which is associated with learning during wakefulness and reactivation during non-rapid eye movement (NREM) sleep. 17 This ensures the redistribution of new information within cortical networks by strengthening synaptic connections. 18

According to the synaptic homeostasis hypothesis, the strengthening of synaptic connections occurs during encoding during wakefulness. 19 Throughout subsequent slow-wave sleep (SWS), synaptic strengthening renormalizes, thereby removing irrelevant and less integrated information and restoring synaptic capacity for new learning. 19

Increased protein synthesis, as required for synaptic strengthening, was first found during NREM. 20 Specifically, this sleep stage is proposed as the period in which short-term plasticity converts into hippocampal long-term plasticity, involving the synthesis of new proteins. 21 Furthermore, it has been reported that sleep elevates cortical messenger ribonucleic acid (mRNA) levels of the genes associated with protein synthesis, which are critical for strengthening existing synapses and building new ones. 22

Finally, electrophysiological markers within the NREM2 and smooth wave sleep stages (NREM 3 and 4, according to the classification of Kales and Rechtschaffen and REM) have also been related to the induction of long-term protein-like plasticity of the hippocampus in the context memory consolidation. 14

Some authors, such as Kempler and Richmond, evaluated the effect of day/night sleep on gross motor learning with uni- and bimanual motor tasks, as well as with whole-body movements in healthy volunteers. 23 The task consisted of bimanual movements, which included sequential combinations of three positions with both arms simultaneously. 23 This test was performed in 70 adults, with participants showing a greater number of precise cycles of the task when repeating the test after sleeping overnight, with no significant change after wakefulness. 23

Another study examined the influence of night sleep on the acquisition of adaptive skills by using bimanual coordination movements. 24 A group of right-handed college students played a shooting video game, which required quick responses to varied visual and auditory stimuli. In this task, players simultaneously manipulated the keyboard with their left hand and the mouse with their right hand. Performance improved along with training and deteriorated after 12 hours of wakefulness. However, it recovered and stabilized after sleeping at night. 24

Authors such as Blischke et al., Malangre et al., and Genzel et al. have pointed out the importance of sleep for the development of gross motor learning, although many studies have reported improvement in gross motor learning after sleeping. 25 26 Other studies have reported stability without additional improvement, arguing the complexity of all aspects related to the process. 27

Sleep deficiency causes impaired motor and neurocognitive performance and involves changes at several systemic levels, such as decreased physical performance, increased mental fatigue, alterations in metabolic and endocrine functions, pain perception, and cognitive and emotional changes. 28

In the brain, lack of sleep has an impact on the release of neurotransmitters, resulting in a decreased ability to learn, store, and retrieve information. 29 30 This could be partially explained by alterations in use-dependent synaptic plasticity. 29 30

Sleep loss inhibits long-term potentiation plasticity of the hippocampus and promotes the induction of short-term potentiation plasticity according to molecular and electrophysiological studies. 31 32 Havekes notes that if long-term potentiation plasticity and cortical excitability are increased after sleep deprivation, the additional learning-induced long-term potentiation plasticity would be less effective, which could be a reason for impaired learning and memory. 33 Furthermore, sleep deprivation reduces the synthesis of proteins' needed to maintain synaptic plasticity, which affects memory consolidation. 33

The human memory is divided into two branches: declarative and procedural memory. Procedural knowledge comprises memories on skills or problem-solving (“know how”). 34 These memories, which may belong to the motor, visual, or even verbal domain, are learned unconsciously, often being referred to as “nondeclarative”. 34 35 Declarative material refers to accessible and conscious memories (“knowing how to understand”). 34 This distinction led to the dual-process hypothesis: 36 the effect of sleep on memory processing would be task-dependent, with the procedural branch being derived from REM 37 and the declarative one linked to NREM. 38

## Review of the Literature

### Materials and Methods

This study is a systematic review of the literature, performed from April to August of 2022, in Bogotá, Colombia, and Sonora, Mexico. A search was conducted on the PubMed and Elsevier databases using the MeSH terms “learning,” “sleep,” “medical in training,” “sleep deprivation,” and “learning in physicians in training”. The inclusion criteria for the selection of articles were: those published in English or Spanish, in the last 22 years (from January 2000 to January 2022). The exclusion criteria were articles in other languages, published before January 2000. The variables considered had an impact on the learning of physicians in training and sleep deprivation.

The articles were independently evaluated. Three of the authors independently read the full text version of 13 articles each. Subsequently, 15 articles were excluded due to not meeting the established criteria.

**Fig. 1:**
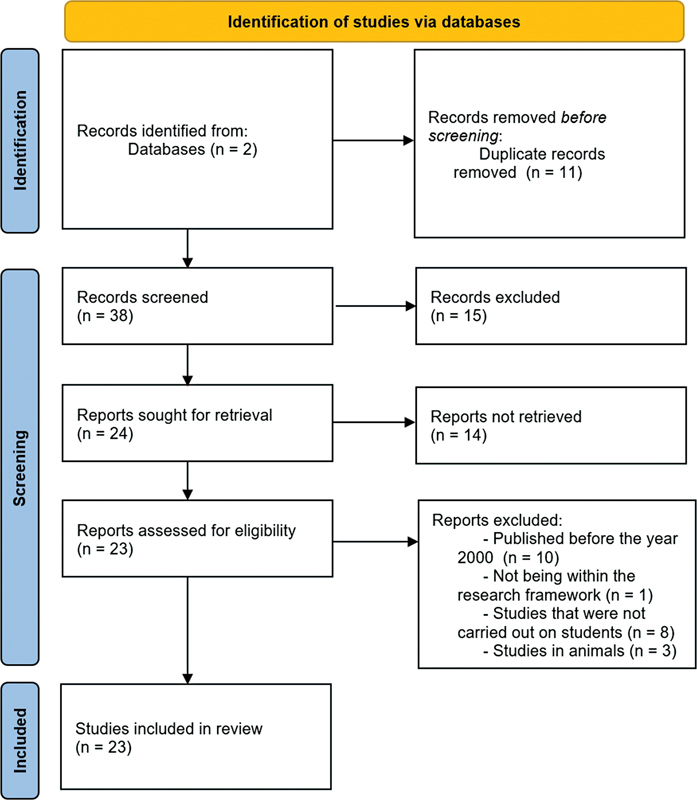
PRISMA flowchart. Disposition of preferred reporting items for this systematic review.

For its preparation, the Preferred Reporting Items for Systematic Reviews and Meta-Analyses (PRISMA) guidelines have been followed for the correct performance of systematic reviews. 39 40 Figure 1 describes the selection process.

## Results

A total of 128 articles were obtained, 31 from the PubMed platform—of which only 22 met the inclusion criteria and 2 were duplicates—and 97 articles from the Elsevier database, with one being selected and 9 duplicates found ( Table 1 ).

**Table 1:** Articles selected according to databases

Databases	Total number of articles	Duplicated	Selected
PubMed	31	2	22
Elsevier	97	9	1

## Discussion

Memory has three phases: registration, short-term memory (minutes-hours), and long-term memory (greater than 24 hours). 36 There are two types of memory: declarative and procedural; the first is learned explicitly, consciously, being divided into episodic memory, time, specific space, and semantic memory of general knowledge; the second one is learned implicitly, that is, unconsciously. 37

Normal human sleep consists of two states: REM and NREM sleep, alternating cyclically. 2 Selective facilitation of declarative memory consolidation has been shown to occur in early SWS-rich sleep, and procedural memory occurs during late REM sleep. 34

Particularly, SWS enhances declarative memories and activities related to the gross motor system, involving neurocortical mechanisms, 12 24 34 while REM sleep preferentially supports aspects of emotional and procedural memory. 17 Studies indicate that this consolidation effect does not occur in all circumstances, instead depending on the type of materials and learning tests. 17

Memory modulation includes numerous endogenous processes activated by hormonal and neural stimuli, in which there is an adaptive function, as well as activation of the hippocampus and the integrative prefrontal cortical-hippocampal network. 13 17

Sleep modulates synaptic connections, which are essential for the formation, integration and reorganization of long-term memory, as well as renewal of the ability learn. 15 The expression of several proteins and genes necessary for synaptic plasticity increases during the first hours of sleep, during which synaptic and cellular homeostasis is restored. 15 19 22

The greatest cognitive/motor demands take place during sleep, requiring more pronounced synaptic stabilization. 14 Learning gains are demonstrated in both implicit and explicit tasks, through generalized sensorimotor skills such as learning specific sequences of a complex motor task. 24 26

Sleep abnormalities are consistently seen in all major brain disorders, both neurological and psychiatric, including schizophrenia, Alzheimer disease, anxiety disorders, and addictions. 28 However, these changes at the neuronal level may be reversible in terms of synaptic plasticity and neuronal loss in different scenarios. 32

Different studies have considered that sleeping for under 6 hours is insufficient. 41 Furthermore, several scales have been used to assess sleep deprivation objectively, such as the Pittsburgh quality sleep index (PSQI) and sleepiness with the Epworth sleepiness scale (ESS) also considered evaluating the correlation between sleep quality and academic performance. 42 Taking into account that health professionals are a population that works for up to 80 hours a week and have flexible hours. This population presents with sleep disorders frequently associated with burnout syndrome, and circadian disorders due to long work hours, shifts, and little rest time. 42

At the pathophysiological level, during sleep deprivation there is a reduction in circulating hormones, as well as a failure in feedback mechanisms, decreased plasticity at the level of the hypothalamus, and regional instability of the network, with increased activation of the adrenal hypothalamic-pituitary-axis, contributing to changes in almost all domains of cognition and affect, predisposing to the development of burnout syndrome and chronic stress. 28 31 43

The consequences of sleep deprivation in healthcare workers increases the risk of complications for the patient at the medical-surgical level, given the many secondary medical errors. 43 44 Sleep deprivation and burnout are correlated with significant medical error. 45 This is more common among attending physicians and those in postgraduate training. 45

The study by Stewart et al. reported that physicians only sleep for 6.5 hours nightly on average, and medical students for less than 7 hours. Those who sleep for less than 6 hours could be more prone to present sleep disorders such as insomnia, delayed phase, decreased efficiency, increased fragmentation, decreased duration, hyperarousal state, and inability to settle down. 42

Different adverse effects have been reported for health personnel, such as changes in mood, depression, anxiety, increased sensitivity to emotional stimuli and stressors, substance abuse, suicidal ideation, and insomnia. 42 43

According to studies, at the epidemiological level, as doctors age, they are more prone to the effects of sleep deprivation, having other repercussions in different aspects of their lives, such as a significant decrease in time with their families due to sleep problems. 42 43

Hafner et al. showed that lower productivity levels and higher mortality risks related to insufficient sleep used to end in economic losses. 46 Insufficient sleep in the population costs about $680 billion of economic output every year in five different Organization for Economic Cooperation and Development (OECD) countries (United States, United Kingdom, Germany, Japan, and Canada). Additionally, it is closely related to factors such as health and wellbeing, which result in large economic losses when affected. 46

Sleep disruption should be recognized as a key factor for memory consolidation and acquisition of new learning. 42 The existing status quo of long periods of work as a routine in health personnel and sleep deprivation being seen as normal or acceptable must be changed. Likewise, the assessment of patients should also be avoided if health personnel have had inadequate sleep. 43

It is necessary to establish a dialogue within the healthcare field, so that strategies and knowledge of sleep deprivation and its consequences can be shared. Government institutions should also establish quality standards to guarantee a better practice with adequate hours of sleep. 43 Furthermore, sleep hygiene must be prioritized in health professionals, as well as sleep diaries and cognitive therapy, and active breaks during working hours. 43

## Conclusion

Quality of sleep is a fundamental factor for the consolidation, processing, and functioning of memory and learning, along with being necessary for adequate work and professional performance. Due to the increased risk of complications, secondary medical errors, and adverse effects associated with the different disorders derived from sleep deprivation, the presence of this condition in health workers has multiple repercussions that must be considered for the wellbeing of both patients and health personnel themselves.

Sleep deprivation should not be considered normal or accepted by health providers, and government entities as well as employers should guarantee optimal working conditions. The wellbeing of health personnel should be comprehensively considered to optimize the services provided to patients.
